# Progressive muscle relaxation reduces migraine frequency and normalizes amplitudes of contingent negative variation (CNV)

**DOI:** 10.1186/s10194-016-0630-0

**Published:** 2016-04-18

**Authors:** Bianca Meyer, Armin Keller, Hans-Georg Wöhlbier, Claudia Helene Overath, Britta Müller, Peter Kropp

**Affiliations:** Institute of Medical Psychology and Medical Sociology, University Medicine Rostock, Gehlsheimer Straße 20, 18246 Rostock, Germany; Center of Integrative Psychiatry, University Clinic of Schleswig-Holstein, Niemannsweg 147, Haus 1, 24105 Kiel, Germany

**Keywords:** Migraine, Contingent negative variation, Progressive muscle relaxation, Cortical preactivation, Migraine prophylaxis

## Abstract

**Background:**

Central information processing, visible in evoked potentials like the contingent negative variation (CNV) is altered in migraine patients who exhibit higher CNV amplitudes and a reduced habituation. Both characteristics were shown to be normalized under different prophylactic migraine treatment options whereas Progressive Muscle Relaxation (PMR) has not yet been examined. We investigated the effect of PMR on clinical course and CNV in migraineurs in a quasi-randomized, controlled trial.

**Methods:**

Thirty-five migraine patients and 46 healthy controls were examined. Sixteen migraineurs and 21 healthy participants conducted a 6-week PMR-training with CNV-measures before and after as well as three months after PMR-training completion. The remaining participants served as controls. The clinical course was analyzed with two-way analyses of variance (ANOVA) with repeated measures. Pre-treatment CNV differences between migraine patients and healthy controls were examined with t-tests for independent measures. The course of the CNV-parameters was examined with three-way ANOVAs with repeated measures.

**Results:**

After PMR-training, migraine patients showed a significant reduction of migraine frequency. Preliminary to the PMR-training, migraine patients exhibited higher amplitudes in the early component of the CNV (iCNV) and the overall CNV (oCNV) than healthy controls, but no differences regarding habituation. After completion of the PMR-training, migraineurs showed a normalization of the iCNV amplitude, but neither of the oCNV nor of the habituation coefficient.

**Conclusions:**

The results confirm clinical efficacy of PMR for migraine prophylaxis. The pre-treatment measure confirms altered cortical information processing in migraine patients. Regarding the changes in the iCNV after PMR-training, central nervous mechanisms of the PMR-effect are supposed which may be mediated by the serotonin metabolism.

## Background

Migraine is one of the most frequent neurological diseases, generated by an interplay of neurological, physiological and psychological factors [1, 2]. It is generally accepted that migraine involves a disturbance of information processing, probably caused by an altered cortical preactivation level [3–5]. One method for examining cortical information processing is the application of event-related potentials, for example the “Contingent Negative Variation” (CNV) [6]. The CNV is a slow cortical event-related potential which develops between two related stimuli. The first serves as a warning stimulus (S1), the second (S2) as an imperative stimulus requiring a motor answer by the subject. The psychological concepts associated with the CNV are motivation, preparation, and attention [7, 8]. Several components can be distinguished: 1) the overall CNV (oCNV), which is the mean amplitude between S1 and S2; 2) the initial CNV (iCNV), which occurs in an interval of 200 ms around the individual amplitude maximum during 550–750 ms after S1; 3) the terminal CNV (tCNV), measured as mean amplitude within the last 200 ms before onset of S2 and finally 4) the habituation coefficient determined by examining the CNV amplitude during the course of a measure with decreasing amplitudes interpreted as habituation and increasing amplitudes interpreted as lack of habituation [7]. Migraine patients exhibit higher CNV amplitudes, especially of the iCNV and the oCNV, as well as a reduced habituation behavior within the interictal interval, which points to a cortical dysbalance [3, 5, 9]. Applying prophylactic treatments, such as betablockers, antiepileptics and even non-pharmacological treatment options like behavioral therapy or physical exercise, CNV parameters tend to normalize. This was interpreted as a possible equalization of the cortical dysbalance [10–13]. Within the non-pharmacological prophylactic treatment options, progressive muscle relaxation (PMR) has not yet been examined in this aspect. PMR is a systematic relaxation technique developed by Edmund Jacobson [14] and is routinely used in migraine attack prevention [15]. The two main principles of this technique are the incompatibility of tension and relaxation and the interaction of muscular and mental levels [16]. PMR has been shown to be potent in the prevention of migraine attacks [17–21]; its effectiveness is comparable to pharmacological migraine prophylaxis [22, 23]. Thus, Grade A was given to PMR by the United States Headache Consortium [24]. However, most of the studies suffer from different deficits. For the most part, they were conducted before introduction of IHS criteria ensuring exact diagnosis. There was often no accurate description of the relaxation technique taught to the participants. In some studies, PMR was taught within one or two sessions [19, 20, 25], which is insufficient according to the recommendations of Bernstein and Borkovec [16]. Thus, in contrast to the persuasion of a high effectiveness for PMR in migraine treatment, the empirical basis is not as solid as expected. Furthermore, little is known about the mechanisms behind the effect evoked by PMR. It was mainly explained by a possible reduction of the autonomic arousal or an enhancement of self-efficacy. For migraine patients, no effects on the cortical level were examined until now. This is surprising regarding the effects of other prophylactic treatment options on CNV-parameters.

Overlooking previous findings concerning CNV and PMR in the context of migraine, the following questions are intended to be answered:

- Can a clinical improvement be achieved by application of PMR?
- As a basis for potential effects of PMR on CNV parameters: can we replicate known differences in the amplitudes of the iCNV, the oCNV, and the habituation coefficient between migraine patients and healthy controls?
- Does PMR have an effect on iCNV amplitude, oCNV amplitude and habituation coefficient?

## Methods

The study conformed to the Declaration of Helsinki and was approved by the Ethics committee of the University Medicine Rostock (ID: A 2011 29).

Overall, four groups were compared: migraine patients and healthy controls with one subgroup each conducting a PMR-training and the remaining participants staying on the waiting-list. Measurements took place before and after the PMR-training as well as 3 months after completion.

Sample size was calculated using the following formula for ANOVA with repeated measures [26] resulting in 21 participants per group and therefore 84 participants altogether.

$$ n=\frac{\lambda_{df;\alpha; 1-\beta }}{\frac{\varOmega^2}{1-{\varOmega}^2}}\cdot \frac{1-r}{p}=\frac{9.63}{\frac{0.06}{1-0.06}}\cdot \frac{1-.06}{3}=20.1 $$

*n*, number of participants; λ, noncentral parameter defining skewness and kurtosis of the alternative hypothesis distribution; α = 0.05; β = 0.80; Ω^2^, effect size; r, correlation between measures; p, number of repeated measures.

To face possible drop out of participants, 52 migraine patients and 50 healthy controls were recruited from September 2012 to November 2013 by newspaper announcements, flyers stored in general medical practices and a newsletter sent to the staff of the University Medicine Rostock. After being informed and examined with structured interviews by neurologists or pain specialists, participants gave informed consent and were quasi-randomly assigned to either an intervention or a waiting-list group. Participants of the waiting-list group were offered to take part in PMR-training after completion of the study. Inclusion criteria were age between 18 and 65 years, absence of chronic somatic or psychiatric diseases (other than migraine in the case of migraine patients) and for migraine patients duration of disease at least 1 year, less than 15 days with migraine per month, less than 10 days with acute medication, no prophylactic treatment during the 3 months before onset of the study. Exclusion criteria were pregnancy or use of drugs. In the course of the study, 7 migraine patients and 4 healthy persons dropped out (largely because of lack of time) and were excluded from analysis; another 10 migraineurs were excluded from analysis because of a migraine attack within 3 days after the CNV-measure. This high rate of excluded patients was unexpected but necessary to insure interpretability of the results regarding the periodicity of the CNV [27]. Neither the dropped out participants nor the excluded patients differed in the pre-intervention measures from the participants who completed the study with the exception of migraine frequency: participants who dropped out showed significantly fewer migraine days and migraine attacks per month.

Overall, four different subject groups were examined in this study. The first (*n* = 16) and the second group (*n* = 19) consisted of migraine patients with and without aura, diagnosed according to the IHS-criteria [28]. The third (*n* = 21) and the fourth group (*n* = 25) consisted of healthy controls without migraine in the personal or family history. All participants were aged between 18 and 63 years. Table 1 shows demographic and clinical characteristics of the sample.

**Table 1:** Demographic and clinical characteristics of the sample

	Migraine PMR (group 1)	Migraine waiting-list (group 2)	Healthy PMR (group 3)	Healthy waiting-list (group 4)	sign. (2-tailed)
*n*	16	19	21	25	
Age (years)	36.4	33.8	37	37,2	0.79 (n.s.)
Male (%)	3 (18.8)	1 (5.3)	5 (23.8)	7 (28)	0.28 (n.s.)
Female (%)	13 (81.2)	18 (94.7)	16 (76.2)	18 (72)	0.28 (n.s.)
Duration of disease (months)	136.5	135.7	–	–	0.98 (n.s.)
Type of migraine (MO/MA)	10/6	14/5	–	–	0.36 (n.s.)
Monthly migraine attacks/days with migraine	3.81/5.5	3.47/5.79	–	–	0.56 (n.s.)/0.74 (n.s.)

*MO* migraine without aura, *MA* migraine with aura, *sign*. significance, *n.s*. not significant

**Fig. 1:**
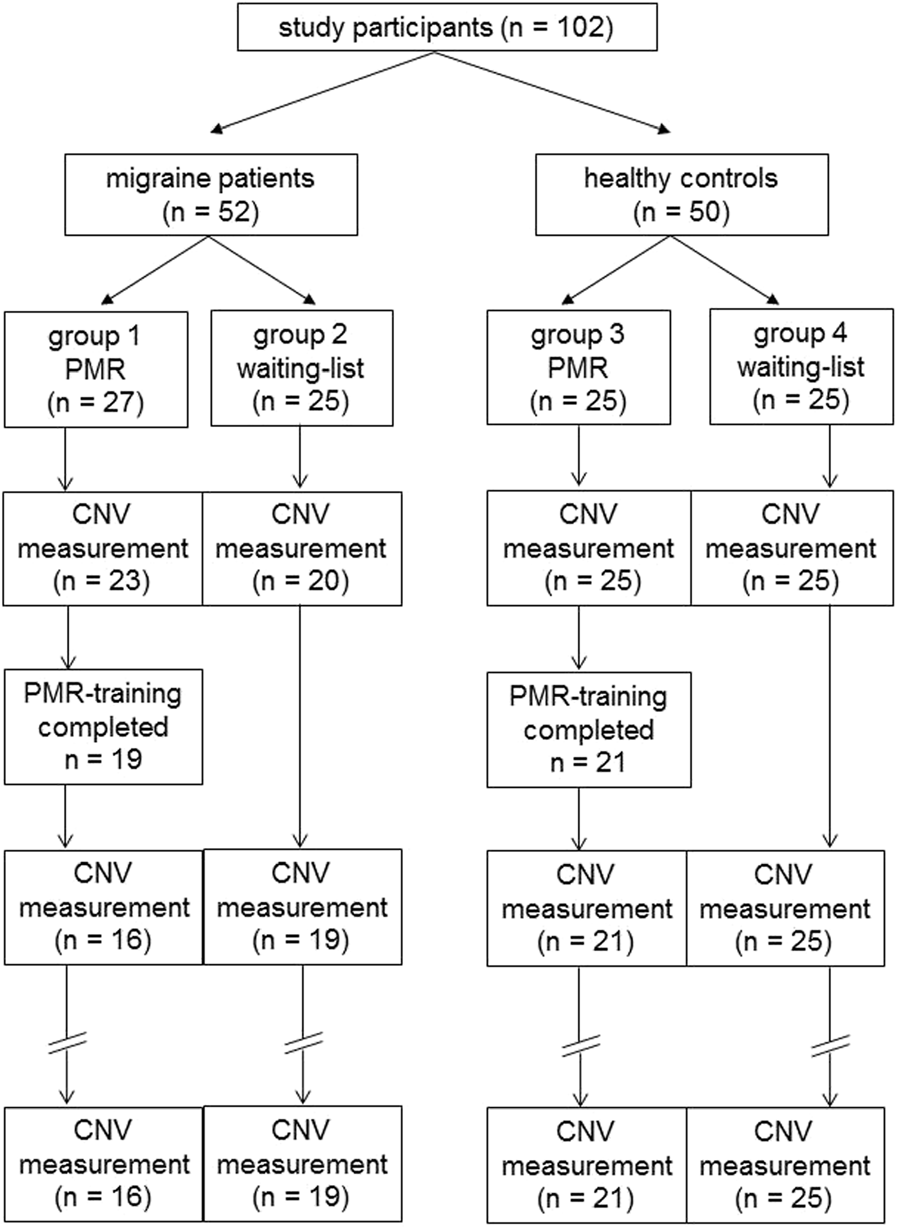
Process of the study for the different groups

One migraine group (group 1, *n* = 16) and one healthy control group (group 3, *n* = 21) conducted a PMR-training program consisting of six weekly sessions. Participants who conducted the PMR-training underwent a CNV-examination before and after the training as well as 3 months after completion. Subjects in the waiting-list groups underwent three CNV-measures at intervals of 4 weeks. Because of the periodicity of the CNV [27], the recordings took place at least 3 days before or after a migraine attack, verified by telephone call after the recording session. All migraine patients kept headache diaries during the course of the study in order to register migraine frequency. Figure 1 gives an overview of the course of the study.

### CNV-recordings

CNV-recordings took place at similar times of day for each subject. The participants were seated in a relaxed position and instructed to focus on a designated spot on the wall to avoid eye movement artifacts. The CNV-measure comprised 32 Go-trials consisting of an acoustic warning stimulus (S1; 1000 Hz, duration 100 ms) and an acoustic imperative stimulus (S2; 2500 Hz, maximum duration 1000 ms). Subjects were asked to press a button immediately after onset of S2, which instantly interrupted the tone. Recordings of each trial started 1000 ms before onset of S1. This period was used for a baseline measurement. The interval between S1 and S2 (interstimulus interval, ISI) was 3000 ms and the recordings ended 1000 ms after S2. Thus, each trial had a duration of 5000 ms. The interval between two trials (intertrial interval, ITI) was randomized between 6 and 10 s. Additionally to these Go-trials, 8 randomly presented NoGo-trials, consisting of a 200 Hz-tone without any answer requested by the subject were added to keep participants in an attentive state [9]. These trials were not analyzed.

The CNV was recorded over Cz according to the international 10-20-System with Ag/AgCl ring electrodes. Linked mastoids were used; the impedances were kept below 10 kOhm. Eye movements were controlled by an electrooculogramm (EOG) with an electrode placed 2 cm below the left eye.

Stimuli presentation was controlled by the program E-Prime, CNV-recording was done with Brain Vision Recorder 1.20 and data analysis was implemented with Brain Vision Analyzer 1.05.

### CNV analysis

The overall CNV (oCNV) was calculated as the mean amplitude between S1 and S2. For iCNV amplitude, the individual amplitude maximum in the interval between 550 and 750 ms after S1 was determined according to Böcker et al. [7]. The mean amplitude in the window of 200 ms around this maximum was defined as the iCNV. The tCNV was defined as mean amplitude in the 200 ms before onset of S2. Additionally, the habituation coefficient was calculated by assigning the Go-trials to 8 blocks and calculating the average amplitude for each block. Using regression analysis, the individual course of the CNV was determined with a positive slope indicating habituation behavior and a negative slope indicating lack of habituation. Data acquisition was conducted unblinded, data analysis was blinded.

With regard to previous studies, only results concerning iCNV, oCNV, and habituation of the iCNV are presented.

### PMR training program

According to Bernstein and Borkovec [16], the PMR-training program included 16 muscle groups that were slightly tensed and thereafter relaxed. Participants were told to focus on the sensations of tension and relaxation and the difference between these two states which results in a deep state of relaxation. They were taught the long PMR-version, followed by successive reductions of the sequences with 7 and 4 muscle groups in the second and third session, respectively. The fourth session involved an envision exercise without muscle tension. The 5th and the 6th sessions comprised conditioned and differential relaxation. Training sessions were conducted in groups of 8 to 10 participants (migraineurs and healthy controls conjoined).

### Statistical analysis

Statistical analyses were conducted using SPSS 21 (Statistical Package of Social Sciences). For clinical data we performed a two-way ANOVA (analysis of variance) with repeated measures to examine the influence of the between-group-factor *intervention* (PMR vs. waiting-list) on migraine frequency. T-tests were performed to find possible differences in the amplitude of the iCNV, the oCNV, and the habituation coefficient between migraine patients and healthy controls in the pre-measure. Lastly, a three-way ANOVA with repeated measures with the between-group-factors *diagnosis* (migraine vs. healthy) and *intervention* was conducted to detect possible changes within the different CNV-components after completion of a PMR-training. For most of the outcome variables the ANOVA requirements of normal distribution, homogenous variances and homogeneous correlations were fulfilled. There were only slight violations for a small number of variables which could be tolerated given the robustness of the ANOVA against these deviations. The significance level was kept at 0.05 with two-tailed testing.

## Results

### Clinical data

Two-way ANOVA with repeated measures revealed significant interactions for time x intervention for both the number of migraine attacks per month (F_time*PMR_ = 4.62; *p* = 0.017) and migraine days (F_time*PMR_ = 3.39; *p* = 0.046), which corresponds to a significant reduction of migraine attacks and days with migraine per month in the group of patients with PMR-training compared to those from the waiting-list. Additionally, a significant time-effect occurred both for migraine attacks (F_time_ = 4.46; *p* = 0.02) and days with migraine (F_time_ = 8.59, *p* = 0.001).

**Fig. 2:**
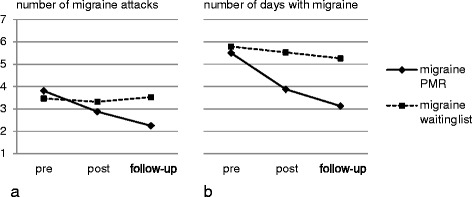
Migraine frequency for both migraine groups in the course of the study for **a** number of migraine attacks and **b** days with migraine

Table 2 displays descriptive data for migraine frequency at all measures; Fig. 2 shows migraine frequency in the study course for both groups.

**Table 2:** Migraine frequency of both migraine groups for all measurement points

		*n*	Number of migraine attacks		Number of days with migraine	
			M	SD	M	SD
1 (pre)	PMR	16	3.81	1.8	5.5	2.77
	Waiting-list	19	3.47	1.61	5.79	2.3
2 (post)	PMR	16	2.88	1.41	3.88	2.28
	Waiting-list	19	3.32	1.77	5.53	3.1
3 (follow-up)	PMR	16	2.25	1.34	3.13	1.93
	Waiting-list	19	3.53	1.98	5.26	2.2

*n* number of patients, *M* mean value, *SD* standard deviation

The decline in migraine frequency corresponds to a reduction of 24 and 41 % for number of migraine attacks from the first to the second and the third recording, respectively. For number of days with migraine, this represents a reduction of 29 and 43 %, respectively.

### CNV-data: comparison of migraine patients and healthy controls

**Fig. 3:**
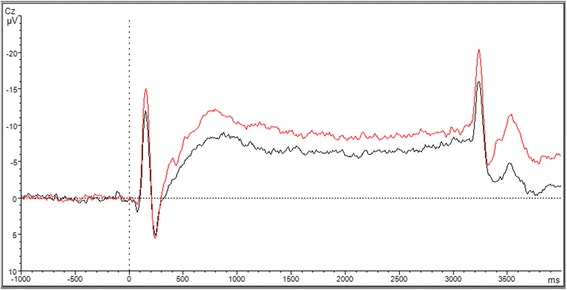
CNV Grand Averages for migraine patients (*red*) and healthy controls (*black*)

Migraine patients exhibited significantly higher iCNV-amplitudes (*t* = −2.63; *p* = 0.006) and oCNV-amplitudes (*t* = −3.39; *p* < 0.001) compared to controls in the first measure. There were no statistical differences regarding the habituation coefficient (*t* = −0.72; *p* = 0.22). Pre-treatment CNV data are presented in Table 3; Fig. 3 shows CNV Grand Averages for migraine patients and healthy controls.

**Table 3:** Pre-treatment CNV data for migraine patients vs. healthy controls

	M migraine patients (*n* = 35) (SD)	M healthy controls (*n* = 46) (SD)	*t*-value (*p*)
iCNV amplitude (μV)	−11.65 (5.65)	−8.62 (4.37)	−2.63 (0.006^**^)
oCNV amplitude (μV)	−8.66 (2.65)	−6.7 (2.51)	−3.39 (<0.001^***^)
Habituation coefficient	−0.05 (1.08)	0.13 (1.11)	−0.72 (0.22)

*μV* microvolt, *M* mean value, *n* number of persons, *SD* standard deviation; **p <0.01, ***p <0.001

### Longitudinal CNV-data: effect of PMR-training on CNV parameters

ANOVA revealed significant main effects of time (*F* = 3.72; *p* = 0.029) and diagnosis (*F* = 5.53; *p* = 0.023) for iCNV amplitudes which represents significantly higher amplitudes for migraine patients compared to controls and a significant change in the course of the study across all groups. Still, a significant interaction effect time x diagnosis x intervention (*F* = 3.39; *p* = 0.039) was found which points to relevant reductions of the iCNV amplitude in the migraine intervention group. With the object of testing for a normalization of this CNV component, groups 1 (migraine PMR) and 4 (healthy waiting-list; representing normal population) were compared at post and follow-up measurement points. There were no significant differences between these groups at measure 2 (*t* = −0.63; *p* = 0.53) nor measure 3 (*t* = −0.73; *p* = 0.47), representing a normalization of the iCNV.

For the amplitudes of the oCNV, ANOVA revealed significant main effects of time (*F* = 11.96; *p* < 0.001) and of diagnosis (*F* = 6.06; *p* = 0.016) showing overall higher oCNV amplitudes for migraine patients compared to healthy participants and a decline of amplitudes across all groups in the course of the study.

For the habituation coefficient there were no main or interaction effects. For a better understanding of this unexpected finding, correlation analyses for the non-intervention groups (groups 2 and 4; migraine waiting-list and healthy waiting-list) over the different measurement points were conducted to estimate the stability of the habituation coefficient across repeated measurements. Although no changes were anticipated in these groups, the analysis revealed correlation coefficients close to zero (r_pre/post_ = −0.004, *p* = 0.98; r_pre/follow-up_ = 0.14, *p* = 0.38; r_post/follow-up_ = 0.004, *p* = 0.98).

**Fig. 4:**
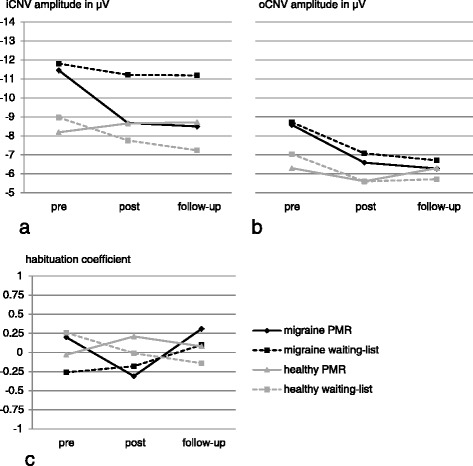
CNV-parameters for all groups in the course of the study for **a** iCNV amplitude, **b** oCNV amplitude and **c** habituation coefficient

**Fig. 5:**
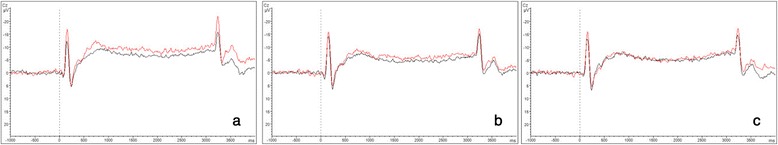
CNV Grand Averages for migraine PMR patients and healthy controls without PMR-training. Migraine PMR patients are displayed in red, healthy controls without PMR are displayed in *black* **a** before **b** after and **c** 3 months after completion of the PMR-training

Table 4 lists descriptive data for the CNV-components for all groups and all measures; Fig. 4 shows CNV parameters in the course of the study for all groups. Figure 5 shows CNV Grand Averages for migraine PMR group and healthy waiting-list group at the pre, post and follow-up measure pointing to approximating CNV curves and thus normalization of the CNV in the migraine PMR group.

**Table 4:** CNV data for all groups at pre, post and follow-up measurement

			Pre-treatment amplitude in μV (SD)	Post-treatment amplitude in μV (SD)	Follow-up amplitude in μV (SD)
iCNV	Migraine	+ PMR	−11.46 (4.79)	−8.68 (3.54)	−8.5 (4.88)
		− PMR	−11.81 (6.4)	−11.22 (4.85)	−11.19 (5.1)
	Healthy	+ PMR	−8.19 (3.25)	−8.66 (4.35)	−8.72 (3.58)
		− PMR	−8.97 (5.18)	−7.76 (5.13)	−7.24 (5.64)
oCNV	Migraine	+ PMR	−8.58 (2.26)	−6.59 (1.93)	−6.28 (2.41)
		− PMR	−8.72 (3)	−7.08 (2.07)	−6.71 (2.55)
	Healthy	+ PMR	−6.3 (2.23)	−5.61 (4)	−6.3 (2.57)
		− PMR	−7.04 (2.73)	−5.6 (2.74)	−5.72 (3.72)
Habituation coefficient	Migraine	+ PMR	0.20 (1.28)	−0.31 (0.94)	0.31 (0.58)
		− PMR	−0.26 (0.87)	−0.18 (0.93)	0.10 (0.87)
	Healthy	+ PMR	−0.03 (1.23)	0.21 (0.85)	0.08 (0.81)
		− PMR	0.26 (1.01)	−0.01 (0.83)	−0.14 (0.82)

*μV* microvolt, *SD* standard deviation

## Discussion

### Clinical efficacy of a six-week PMR-training

Our study confirmed that PMR exhibits a substantial effect on migraine reduction. After a 6-week treatment program with progressive relaxation according to Bernstein and Borkovec [16], migraine frequency decreased significantly. The significant interaction terms of time and intervention for both migraine attacks and days with migraine demonstrate that migraine patients benefit from a regular PMR-training having a lower migraine frequency compared to those without relaxation treatment.

Thus, we could show a specific migraine-reducing effect due to the elaborate acquisition of PMR relaxation technique which could not be inferred in such a distinct way from previous studies [17–21]. As shown in other studies, migraine frequency even decreased after completion of the treatment [23]. PMR was suggested to operate by enhancing self-efficacy [23]. With every successful relaxation exercise migraine patients may develop growing confidence in their own coping abilities. It can be expected that for migraineurs, the feeling of being able to control one’s own relaxation state is helpful in dealing with the disease and reinforces control expectancy. Actually, there is growing interest in the role of expectancy in the context of migraine treatment. Recent findings showed the meaning of patients’ expectations for the amount of treatment efficacy [29]. Thus, these expectations should be taken into account in the context of behavioral migraine prophylaxis.

### CNV data: differences between migraine patients and healthy controls in the CNV parameters

As expected, migraine patients exhibited significantly higher amplitudes in the iCNV and oCNV pre-treatment measure which is in line with other studies (e.g. [9]). These findings were mainly interpreted to be caused by a lower cortical preactivation level and to concern attention allocation [5, 9]. From a theoretical point of view, one explanation was seen in the ceiling theory [30, 31] which assumes that for evoked potentials, cortical reactivity is reduced after having reached a ceiling point which initiates a habituation response. According to the ceiling theory, a lower preactivation level in migraine patients would cause a delayed or missing habituation because the ceiling would be reached later than in healthy people. In this context, overall higher amplitudes of evoked potentials in migraine patients are seen as a consequence of the missing or reduced habituation.

Unexpectedly, we did not find significant differences in the habituation behavior between migraine patients and controls although this seems to be a quite robust finding in other studies for different modalities [9, 32]. As the mean values for migraine patients and for healthy controls present the expected (albeit not significant) differences with migraineurs showing a negative habituation coefficient (habituation deficit) and controls a positive habituation coefficient (habituation), this has to be ascribed to the low number of participants and thus the low power. Still, not all studies could show a CNV habituation deficit of migraine patients in the past. Mulder et al. found similar habituation processes in migraineurs and healthy controls; but a number of methodological and recruitment-associated reasons limit the comparability to our results [33]. In VEP studies, Omland et al. [34] could not observe any lack of habituation in migraine patients which they supposed to be largely an effect of blindedness; past trials with findings of habituation deficits have not been blinded. However, especially in the CNV, there may be many alternative, possibly more important factors than blindedness, which influence habituation behavior [35]. Restrictedly, the small sample size of the current study doesn’t offer any further analysis of this point.

Even if our data do not confirm all presumptions of the ceiling theory, we found and altered information processing which presumably can be explained by an altered preactivation level.

### Longitudinal CNV data: effect of PMR-training on CNV parameters

For iCNV amplitude, ANOVA revealed a significant interaction of time, diagnosis and intervention which indicates that the greatest changes of iCNV amplitudes appear within the migraine PMR group. A *t*-test of mean differences for migraine patients with PMR and healthy controls without PMR who are supposed to represent normal population revealed no significant iCNV differences, neither at measure 2 nor at measure 3. This result points to a normalization of iCNV amplitudes. For oCNV amplitudes, we found significant main effects of time and diagnosis illustrating an overall higher negativity of oCNV amplitudes in migraine patients irrespective of the intervention. The main effect of time represents considerable changes of the oCNV in all groups. For the habituation coefficient no main or interaction effects could be found. Additionally, there were no correlations between the different measurements for the non-intervention groups.

The longitudinal findings are partly in line with an amount of other studies. For the iCNV amplitude, we could show a normalization as it was seen after application of other prophylactic treatment options [10–13]. This is interpreted as an effect of PMR on the cortical level. A mediating factor may be the neurotransmitter serotonin which was shown to be reduced in migraine patients in the interictal interval [36]. Also, serotonin is known to influence the preactivation level [36]; so a dysregulation of the serotonin level is expected to cause a dysbalance of the cortical preactivation level. However, there are no studies examining the effect of relaxation techniques on the serotonin level, so a missing link persists. At least one review indicates an effect of relaxing massages on serotonin metabolism [37], leading to the hypothesis that PMR-training may exhibit cortical effects by balancing the serotonin metabolism. In this sense, a subsequently more balanced preactivation level may help to prevent migraine attacks.

For the amplitude of the oCNV, no PMR-specific changes were found. The main effect of time in the absence of any interaction effect demonstrates an overall decline of the amplitudes in the course of the study whereat migraine patients exhibited higher amplitudes at any measure (main effect of diagnosis). Thus, the oCNV seems to be unaffected by PMR but to systematically decline in repeated measures. One possible explanation could be the existence of long-term habituation processes [38], a phenomenon which is not well examined so far and has to be interpreted with caution.

A striking result of the current study is the absence of main or interaction effects concerning the habituation coefficient which is in opposition to other studies that found the habituation coefficient to be influenced by prophylactic treatment [11–13]. Since in most studies the same methods of data acquisition and analysis were used, no methodological differences could explain the discrepancy. Presumably, the differences can at least partly be explained by the low number of participants. The aspired sample size could not be reached which results in low power. This difficulty is aggravated by the high variability of the habituation coefficient, visible for example in the high standard deviation in the first measure of the migraine PMR group. However, in our study, habituation behavior all in all was found highly variable as can be seen in low correlations across the repeated measurements for the non-intervention groups. Actually, there is no study examining the reliability of the habituation coefficient of the CNV. Instead, some authors already emphasized the high variability of evoked potentials and their sub-components in migraine patients [34, 39]; others found low reliability values in VEPs and emphasized the necessity of (large) group analyses to interpret habituation indexes [40]. Thus, habituation behavior may be a variable dimension with a high sensitivity to constantly changing external (e.g. noise, odor) or internal (e.g. motivation, tiredness) factors that could not sufficiently be controlled for in our study due to the small sample size.

Study limitations of the current investigation are primarily the low number of participants due to the periodicity of the CNV and the resulting exclusion of several measures. The aspired number of participants could not be reached which is supposed to have contributed to the unexpected results concerning the habituation coefficient. Furthermore, the differing participant recruitment mode with patients not being recruited from specialized headache ambulances, but from general population, potentially limits the comparability to other investigations. However, this presumably enhances the generalizability of our findings to normal migraine population [40]. In future studies, more participants should be included, possibly with a multicenter approach. A longer follow-up period may be favorable to examine the stability of the effects found.

## Conlusions

In summary, this study shows a clinical effect of regular PMR application on migraine frequency. Additionally, a normalization of the iCNV in migraine patients after PMR-training indicates that PMR is operating not only on the psychological, but also on the electrophysiological level. A clinical implication of the results may be that patients are given a more detailed explanation of the effects of PMR on the electrophysiological level, which might fill a perceived gap between a somatic disorder and a behavioral intervention.
